# Design and fabrication of electrochemical sensor based on NiO/Ni@C-Fe_3_O_4_/CeO_2_ for the determination of niclosamide

**DOI:** 10.1038/s41598-024-58319-w

**Published:** 2024-03-30

**Authors:** Setayesh Darvishi, Ali A. Ensafi, Kimia Zarean Mousaabadi

**Affiliations:** 1 Department of Chemistry, Isfahan University of Technology, Isfahan, 84156-83111 Iran; 2 Department of Chemistry & Biochemistry, University of Arkansas, Fayetteville, AR 72701 USA

**Keywords:** Niclosamide, Electrochemical sensor, MOF-derived nanocomposite, Metal-oxide nanoparticles, Green synthesis, Health care, Chemistry, Nanoscience and technology

## Abstract

In this study, we aimed to enhance and accelerate the electrochemical properties of a glassy carbon-based voltammetric sensor electrode. This was achieved through the modification of the electrode using a nanocomposite derived from a metal–organic framework, which was embedded onto a substrate consisting of metal oxide nanoparticles. The final product was an electrocatalyst denoted as NiO/Ni@C-Fe_3_O_4_/CeO_2_, tailored for the detection of the drug niclosamide. Several techniques, including FT-IR, XRD, XPS, FE-SEM, TEM, and EDS, were employed to characterize the structure and morphology of this newly formed electroactive catalyst. Subsequently, the efficiency of this electrocatalyst was evaluated using cyclic voltammetry and electrochemical impedance spectroscopy techniques. Differential pulse voltammetry was also utilized to achieve heightened sensitivity and selectivity. A comprehensive exploration of key factors such as the catalyst quantity, optimal instrumental parameters, scan rate influence, and pH effect was undertaken, revealing a well-regulated reaction process. Furthermore, the sensor's analytical performance parameters were determined. This included establishing the linear detection range for the target compound within a specified concentration interval of 2.92 nM to 4.97 μM. The detection limit of 0.91 nM, repeatability of 3.1%, and reproducibility of 4.8% of the sensor were calculated, leading to the observation of favorable stability characteristics. Conclusively, the developed electrochemical sensor was successfully employed for the quantification of niclosamide in urine samples and niclosamide tablets. This application highlighted not only the sensor’s high selectivity but also the satisfactory and accurate outcomes obtained from these measurements.

## Introduction

Niclosamide, a medication that fights worms (called an "anthelmintic"), has been essential in getting rid of tapeworm infections. This has had a major impact on people's health. The World Health Organization (WHO) says this worm-killing medicine can also be used to control snails that spread schistosomiasis, a parasitic disease^1^.

Niclosamide kills tapeworms effectively, making it a valuable treatment for tapeworm infections. However, it doesn't work against other worms like pinworms or roundworms. The right dose depends on the type of worm and the patient's age, and comes in chewable tablets for easy use^2^. It works by targeting two crucial functions of the worms: their energy production (oxidative phosphorylation) and their sugar absorption. By disrupting these essential processes, Niclosamide effectively weakens and eliminates the worms responsible for the infection^3^.

While niclosamide has a wide range of applications, it can be toxic to some aquatic organisms, such as fish and invertebrates, causing reduced growth, impaired reproduction, or even death. Additionally, when used for extended periods, it can harm plants by inhibiting their growth, altering their nutrient uptake, or changing the soil microbial communities^4,5^. Niclosamide is crucial for many treatments, but it can cause side effects like chest tightness, dizziness, and fatigue. To ensure its safe use, we urgently need a better way to track and measure its levels in the body. This will help us understand and minimize the risks associated with this valuable drug^3,6^.

Traditionally, high-performance analytical techniques such as GC/MS, HPLC, and LC–MS have constituted the mainstay of cestode research, offering high resolution separation, ultrasensitive quantification, and versatile analytical capabilities. However, their substantial financial investment, demanding technical expertise, and inefficient throughput limitations significantly constrain the implementation of simpler, swifter, and more cost-effective analytical methodologies. Therefore, the development of innovative tools is imperative to overcome these inherent limitations and facilitate a paradigm shift in cestode analysis^6^. Electrochemical sensors have captivated researchers with their efficiency, simplicity, and affordability. But their true potential lies in their sensitivity, a quality directly linked to the peak current value. Optimizing the electrode surface is the magic bullet for unlocking this potential, paving the way for high-performance sensors^5,7^.

The intrinsic electrochemical sensing capabilities of unmodified electrodes are often hampered by slow reaction kinetics, inadequate sensitivity, and high overpotentials. These limitations can be effectively addressed through the strategic modification of the electrode surface with materials like metal oxides and metal–organic frameworks (MOFs), leading to demonstrably improved sensing performance^7,8^. Recent advancements in nanostructured metal oxides have yielded highly selective and cost-effective electrochemical sensors. By tailoring the morphology, size, and composition of these metal oxide nanoparticles (MO NPs) through versatile synthesis methods, scientists have unlocked a diverse range of electrochemical properties. This fine-tuning empowers the MO NPs to exhibit enhanced catalytic activities, significantly boosting sensor performance^8,9^. Within the diverse landscape of metal oxides, nanoscale cerium oxide (CeO_2_) has emerged as a particularly noteworthy material due to its unique properties. These include facile and reversible redox behavior (Ce^4+^ ↔ Ce^3+^), enhanced surface roughness, and the presence of oxygen vacancies ^9^. Moreover, the unique properties of magnetite (Fe_3_O_4_) nanoparticles, in particular their room-temperature electrical conductivity due to electron hopping interactions between Fe^2+^ and Fe^3+^ ions, have positioned them as a valuable tool in electrochemical sensing. Their widespread adoption in electrode modification highlights their versatility in diverse analyte detection applications^10^. To achieve enhanced electrocatalytic efficiency and significantly amplified oxidation reactions, the authors implemented modifications to create multicomponent composites, moving beyond the usage of individual cerium oxide (CeO_2_) and iron(III) oxide (Fe_3_O_4_). These modifications exploit the crucial redox behavior of each oxide (Ce^3+^/Ce^4+^ and Fe^2+^/Fe^3+^) and capitalize on synergistic interactions between them, leading to the observed improvements in sensitivity and efficiency^9,10^.

Metal–organic frameworks (MOFs) are a class of crystalline materials featuring a highly ordered porous structure, large surface area, and customizable chemical properties^10^. These hybrid materials, combining organic and inorganic components, were first conceived by Yagi et al. in the mid-1990s^7^. MOFs have found widespread application in catalysis due to their accessible metal sites and reactive organic linkers. However, their intrinsic limitations in electrical conductivity and stability under electrochemical conditions restrict their potential in areas like electrocatalysis and energy storage. Emerging efforts focus on tailoring MOFs by generating MOF-derived materials with controlled size, shape, and structure, effectively inheriting the advantageous features of the parent MOF while overcoming its limitations. This holds promise for unlocking wider applications of these versatile materials in addressing critical needs^10,11^. The exceptional ability of metal–organic frameworks (MOFs) to generate well-defined nanostructures has facilitated their recent application in the synthesis of advanced transition metal oxide electrodes. Thermal calcination of MOF precursors offers a rapid route to fabricate carbon-coated metal oxide composites. This metallic/metallic oxide@carbon composite presents several compelling advantages, including enhanced catalytic activity due to the accessibility of active metal sites, tunable pore volume for optimized molecular transport, and a well-ordered architecture that promotes efficient catalytic processes^8^.

The selection of a synthesis method is paramount in defining the morphology, dimensions, and physicochemical characteristics of nanoparticles. Conventional physical approaches are often hampered by heterogeneous particle size distributions and prohibitive equipment costs or potentially hazardous reactants. In contrast, chemical synthesis utilizing bioactive reagents has emerged as a compelling alternative, offering economic viability, environmental sustainability, expeditious protocols, and facile implementation. Demonstrating this principle, we have generated cerium oxide nanoparticles by employing an extract derived from Musa sapientum peels^11^. Ananda et al. used a novel method—synthesizing erbium-doped copper oxide using banana extract—to create a highly sensitive electrochemical nitrite sensor. This new material, attached to a glassy carbon electrode, showed superior performance compared to the bare electrode, with both higher current and lower energy requirements for nitrite detection. Further analysis confirmed the successful synthesis and properties of the erbium-doped copper oxide nanoparticles. Moreover, this material exhibited remarkable photocatalytic activity, degrading nearly all Methyl Red dye under visible light. Studies revealed the optimal conditions for this process, and the material remained stable and effective even after repeated use. These findings suggest that erbium-doped copper oxide holds great promise for both nitrite sensing and dye degradation applications^12^. Zarean et al*.* developed a novel electrochemical sensor to accurately measure methotrexate, a vital cancer treatment drug. Built using NiO/Ni@C and PINA layers on a carbon electrode, it offers exceptional sensitivity and reliability. Extensive characterization revealed the material properties, while optimization fine-tuned its performance. This optimized sensor boasts an impressive detection limit of 7.2 nM, consistent results, and resistance to interference from other substances. It even proved effective in real-world samples like blood plasma^8^. Miri et al*.* investigated the potential of cerium oxide nanoparticles (CeO_2_-NPs) synthesized using Musa sapientum peel extract. They explored their cytotoxic effects, UV protection capabilities, and photocatalytic activity. The synthesized nanoparticles were characterized using Raman spectroscopy, Powder X-ray Diffraction (PXRD), Fourier-Transform Infrared spectroscopy (FT-IR), Transmission Electron Microscopy (TEM), Field Emission Scanning Electron Microscopy (FESEM), and Energy-Dispersive Spectroscopy (EDX). FESEM analysis revealed their size range to be between 4 and 13 nm. Cytotoxicity evaluations on lung (A549) cell lines demonstrated non-toxic behavior at concentrations below 500 μg/mL. Notably, the synthesized CeO_2_-NPs exhibited an estimated Sun protection factor (SPF) of ~ 40. Furthermore, their photocatalytic activity was assessed against AO7 dye under visible light illumination. The results were promising, with 81.7% degradation of AO7 achieved within 180 min^11^. Jayasmita et al*.* achieved a breakthrough in electrocatalytic seawater splitting by designing a novel cathode material—a composite of carbon nanospheres with copper oxide and cobalt oxide (CuO/Co_3_O_4_). This material exhibited exceptional performance, requiring minimal energy (overpotential of 73 mV at 10 mA/cm^2^) and facilitating efficient proton transfer (Tafel slope of 58 mV/dec), while remaining stable and structurally intact over extended testing. The success lies in the synergistic interaction between the metal oxides, which accelerates the crucial proton-electron transfer at the active sites. Furthermore, the carbon nanosphere support plays a crucial role by maximizing the available surface area for the reaction and enhancing the material's overall stability^13^. Furthermore, Tata Sanjay et al*.* used architecting hierarchal nanostructure for electrochemical improvement. Hierarchical nanostructures with improved electrode performance, ample active sites, unique electronic states, and enhanced stability and conductivity are gaining significant attention. This work reports the design of a novel Zn_3_V_2_O_8_/P-rGO nanocomposite using a combined hydrothermal and ultrasonication process. This multicomponent material, featuring transition metal vanadates and 2D graphene, offers several advantages. Electrochemical analysis employing cyclic voltammetry and DPV revealed a remarkably low limit of detection (LOD) of 0.0067 μM and high sensitivity for the analyte detection. The sensor also demonstrated excellent selectivity, reproducibility, and stability. Importantly, its effectiveness was validated in real samples like human blood serum, urine, and wastewater, highlighting its potential for environmentally friendly analyte monitoring^14^.

This study introduces a simple, green, and cost-effective method for creating a new sensor (NiO/Ni@C-Fe_3_O_4_/CeO_2_/GCE) for electrochemical analysis of Niclosamide using Differential Pulse Voltammetry (DPV). The cooperation between NiO/Ni@C and Fe_3_O_4_/CeO_2_ materials, rich in active sites, porous structure, and high conductivity, leads to excellent repeatability, good sensitivity, strong selectivity, long-term stability, and high resistance to interference. These results demonstrate the sensor's suitability for sensitive Niclosamide detection.

## Experimental

### Synthesis of CeO_2_ and Fe_3_O_4_ NPs

CeO_2_-NPs were synthesized by following method in previous literature^11^. The dried and powdered peel of Musa sapientum fruit was extracted using water as a solvent in a 1:10 ratio, employing the soaking method. The resulting mixture was then filtered through Whatman^®^ Filter Paper, and 10 mL of the filtrate was diluted with 40 mL of distilled water. Subsequently, 50 mL of a 0.05 M cerium nitrate solution (Ce(NO_3_)_3_.6H_2_O, Merck) was added to this solution. The mixture was stirred at 70 °C for 4 h. The resulting solution was subsequently dried at 90 °C, and the residue underwent calcination processes at temperature 400 °C. The temperature was increased at 4 °C per minute until the desired temperature was reached and held for 2 h. The CeO_2_-NPs were obtained in the form of yellow powder. Additionally, to synthesize cerium oxide/iron oxide, a procedure like that used for cerium oxide synthesis was employed. The difference lies in the use of a mixture of cerium nitrate and iron chloride salts in a 1:1 molar ratio. The resulting product, Fe_3_O_4_/CeO_2_, was obtained in the form of a brown powder.

### Synthesis of NiO/Ni@C

Ni-MOF was produced using the methods described in earlier literature^8^. To summarize, a solution was prepared by dissolving 1.4 mmol of Ni(NO_3_)_2_·6H_2_O, 0.70 mmol of trimesic acid, and 1.50 g of PVP in a mixed solvent consisting of ethanol, DMF, and water in a 1:1:1 ratio. This solution, which had a light green colour, was achieved by magnetic stirring. Subsequently, 30 mL of this well-mixed solution was transferred into a 50 mL Teflon-lined autoclave and subjected to heating at 150 °C for 10 h. The resulting green sediment underwent three rounds of centrifugation using a mixture of DI water and ethanol in a 1:1 ratio and was subsequently dried in a vacuum drying oven at 80 °C for 12 h. To transform Ni-MOF into NiO/Ni@C, the green powder was introduced into a tube furnace under an N_2_ atmosphere and heated to 450 °C at a ramp rate of 1 °C per minute, maintaining this temperature for 2 h. Ultimately, the final product obtained was the NiO/Ni@C black powder.

The plant collection and use were in accordance with all the relevant guidelines.

### Synthesis of NiO/Ni@C-Fe_3_O_4_/CeO_2_ nanocomposite

Ni/NiO@C and Fe_3_O_4_/CeO_2_ powders were mixed in equal amounts (1:1 mass ratio) and sonicated for 2 h. Finally, the obtained precipitate was dried at room temperature. The dried product was then dispersed in solution to prepare a 1 mg/mL suspension for electrochemical characterization.

## Results and discussions

The essential components found in the peel of *Musa sapientum* fruit, play a crucial role in acting as both reductive agents and stabilizers for cerium ions. This facilitated an oxidation reaction involving the conversion of Ce^3+^ to Ce^4+^, utilizing the abundant phenolic and sugar compounds present in the extract of *M. sapientum*. The resulting Ce(OH)_4_ transformed into CeO_2_ nuclei through dehydration, leading to the creation of spherical nanoparticles^11^.

### Fourier transform infrared spectroscopy analysis (FTIR)

**Figure 1:**
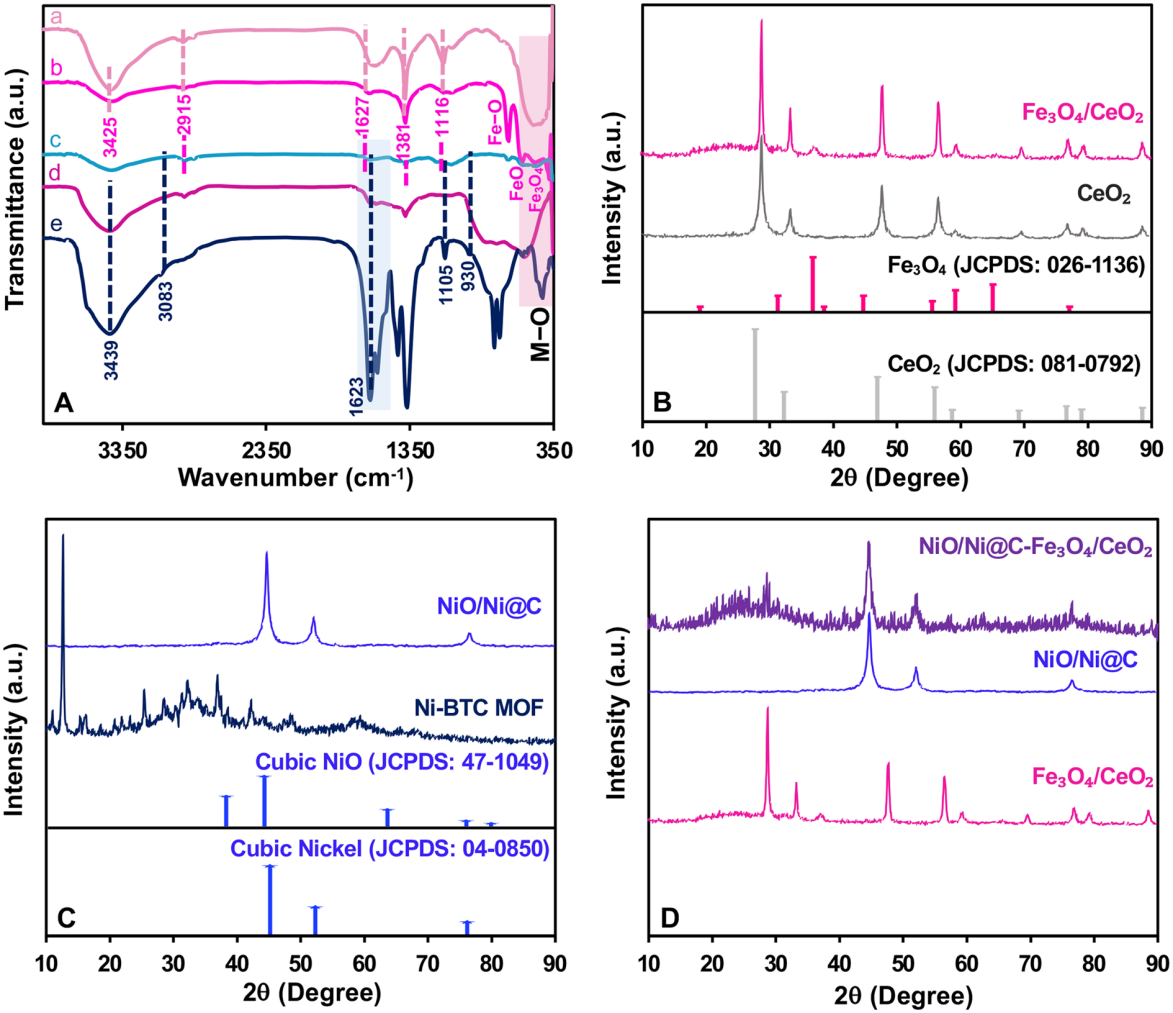
(**A**) FT-IR spectra and (**B**–**D**) XRD patterns of CeO_2_ (a), Fe_3_O_4_/CeO_2_ (b), NiO/Ni@C-Fe_3_O_4_/CeO_2_ (c), NiO/Ni@C (d), and Ni-BTC(e)_._

Figure 1A displays the FTIR spectra for CeO_2_, Fe_3_O_4_/CeO_2_, Ni-BTC, NiO/Ni@C and NiO/Ni@C-Fe_3_O_4_/CeO_2_ nanocomposite. As can be seen, the distinct adsorption bands situated below 600 cm^−1^ indicated the formation of metal-oxide (M–O) (a,b) bonds. Additionally, a strong band at 668 cm^−1^ was assigned to the stretching mode of Fe–O bonds (b). The sample exhibited distinctive absorption bands at 566 cm^−1^, signifying the stretching vibrations of iron(II) oxide, and at 442 cm^−1^ (a,b), which could be associated with the bending vibration mode of Fe_3_O_4_^15^. The presence of two minor absorption peaks at 1116 and 1381 cm^−1^ was attributed to the presence of a residual nitrate group in the structure. Three absorption bands at 3425, 2915, and 1627 cm^−1^ could be ascribed to the stretching and bending vibrations of surface hydroxyl or adsorbed water, along with carbonation on the surfaces of the NiO–CeO_2_ nanostructures, respectively^16^. Moreover, the primary peaks at approximately 1465, 1577, and 1623 cm^−1^ within the Ni-BTC spectrum are related to the presence of Ni ions coordinated with the −COO functional group in the of 1350–1650 cm^−1^ range. Furthermore, 1623, 1105, and 930 cm^−1^ bands correspond to the vibrational frequencies of C=O, C–N, and N–CHO resulting from Ni coordination with DMF molecules. The broad peak at 3439 cm^−1^ corresponds to the stretching vibration of O–H from adsorbed water and coordinated water in the Ni-MOF (e) structure. The stretching vibration peak of C–H emerges at 3083 cm^−1^. In the NiO/Ni@C (d) and Fe_3_O_4_/CeO_2_ (b) spectra, the intensity of these peaks diminish due to the conversion of Ni-MOF (e) to NiO–Ni@C (d), resulting in the removal of the organic ligand from the structure^8^. This analysis affirms the successful formation of NiO–Ni@C-Fe_3_O_4_/CeO_2_ (c).

### X-ray diffraction (XRD) analysis

XRD analysis was conducted to determine the crystalline structure of CeO_2_, Fe_3_O_4_/CeO_2_, NiO/Ni@C, and NiO/Ni@C-Fe_3_O_4_/CeO_2_ in the range of 2θ = 10–90°. The identified diffraction peaks for CeO_2_ and Fe_3_O_4_ matched with specific JCPDS card numbers (08-0792 and 026-136, respectively), as shown in Fig. 1B. The distinct and sharp diffraction peaks of both Fe_3_O_4_ and CeO_2_ were observed at 2θ = 29.05, 33.2, 47.55, 56.40, 59.25, 69.80, 76.70, and 88.85° corresponding to (111), (200), (220), (311), (222), (400), (331), (420) and (422) of CeO_2_ crystallographic planes, respectively indicating its cubic fluorite structure^16^. Additionally, minor peaks seen at 2θ values of 18.15, 28.70,37.10, 44.90, 56.45, 59.30, 65.59, and 76.90° attributed to the (111), (220), (311), (400), (422), (511), (440) and (533) lattice planes of cubic Fe_3_O_4_^17^. The intensity of the peaks related to the Fe_2_O_3_ phase is lower due to its lower amount in the final compound than CeO_2_. Moreover, the XRD data provides valuable insights into the structural features of materials through parameters like crystallite size ($D =\frac{K\lambda }{\beta cos\theta }$), dislocation density ($\updelta =\frac{1}{{D}^{2}}$), and micro strain ($\varepsilon =\frac{\beta }{4tan\theta })$ ^18^. In these equations, *D* represents the average crystallite size, *K* is the Scherrer constant (usually approximately 0.9),* λ* denotes the wavelength of X-rays or neutrons, *β* signifies the full width at half maximum (FWHM) of the diffraction peak, and *θ* is the Bragg angle. We calculated these parameters for both CeO_2_ and Fe_3_O_4_/CeO_2_ based on the main peaks of CeO_2_. Crystallite size, dislocation density, and micro strain were calculated as 14.1 nm, 5.1 × 10^–3^ nm^−2^, and 5.8 × 10^–3^, respectively, for CeO_2_, and 19.5 nm, 2.7 × 10^–3^ nm^−2^, and 3.9 × 10^–3^, respectively, for Fe_3_O_4_/CeO_2_. These parameter alterations can be attributed to the introduction of Fe_3_O_4_, influencing the crystalline structure of CeO_2_. The larger crystallite size in the composite material suggests enhanced crystal growth, while the reduced strain and dislocation density indicate a more stabilized and ordered lattice structure compared to pure CeO_2_. Besides, these changes are not notably significant due to Fe_3_O_4_/CeO_2_ is two phase composite material.

Figure 1C presents information regarding the XRD patterns of Ni-BTC and NiO/Ni@C. The XRD pattern of Ni-BTC displays distinct peaks that unveil the Ni-BTC´s pure phase and crystalline arrangement. The peak observed at 2θ value of 12.67° confirmed the presence of MOF structure. Post-calcination and the conversion of Ni-BTC to NiO/Ni@C, the surface roughness of Ni-BTC increases, leading to a reduction in sphere diameter. The peaks observed at 2θ values of 44.6, 52.0, and 76.52° match with the cubic NiO's lattice planes of (111), (200), and (220) respectively (JCPDS 47-1049)^8^.

NiO/Ni@C or NiO@C was produced through a two-step heating procedure using Ni-MOF (Nickel-Metal Organic Framework), denoted as Ni–BTC. Initially, Ni–BTC was created via solvothermal synthesis, and subsequently, it was heated in a nitrogen environment to yield C@Ni microspheres. These C@Ni microspheres were further subjected to annealing in an oxygen-rich environment to generate NiO/Ni@C or NiO@C^19^. Figure 1D demonstrates the presence of peaks resulting from NiO/Ni@C and Fe_3_O_4_/CeO_2_ patterns in the NiO/Ni@C-Fe_3_O_4_/CeO_2_ pattern has appeared with lower intensity, which is due to the formation of weak bonds between them, and it also confirms the composite formation of the final compound.

### X-ray photoelectron spectroscopy (XPS) analysis

**Figure 2:**
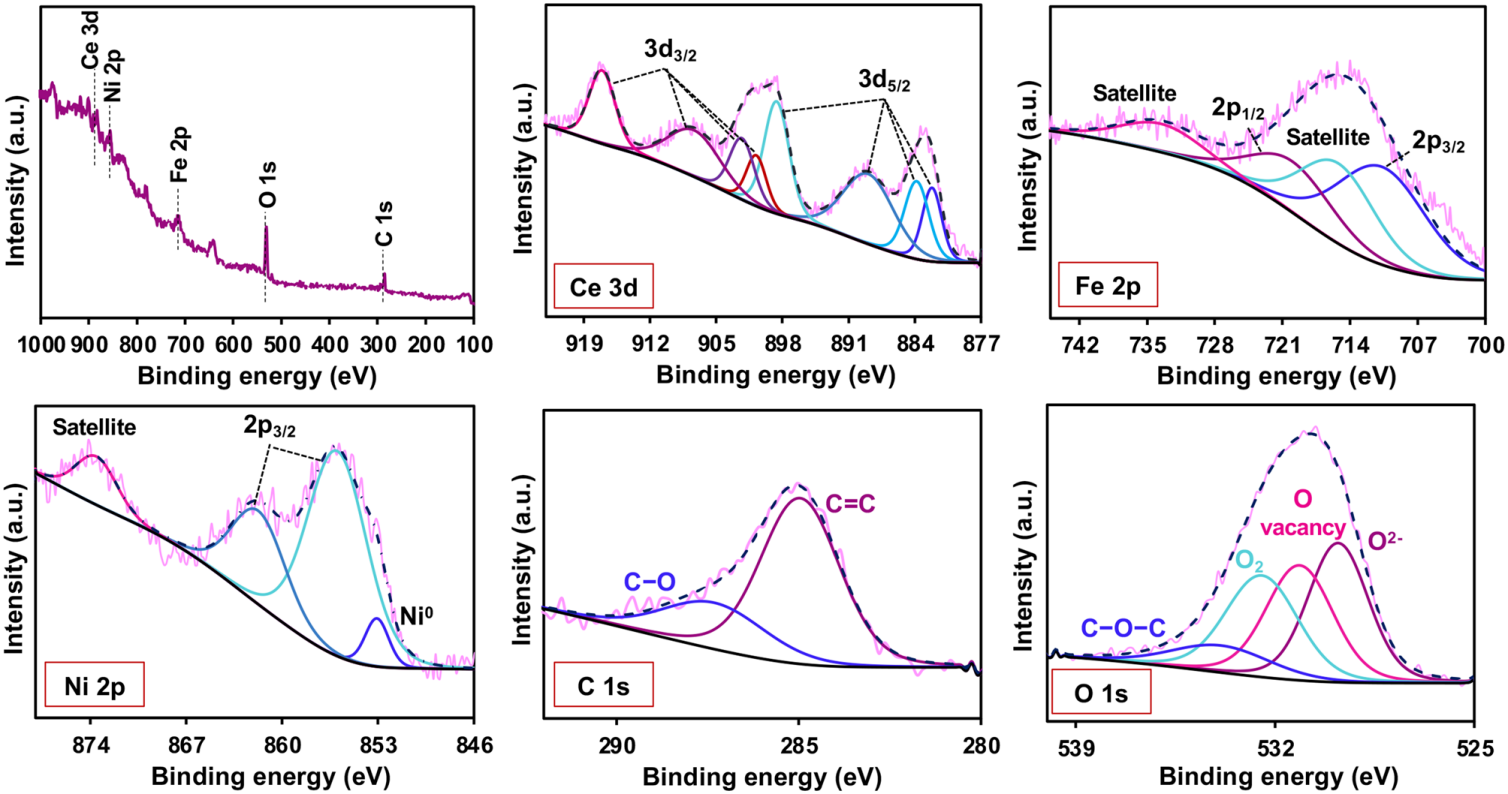
XPS spectrum of NiO/Ni@C-Fe_3_O_4_/CeO_2_ and high-resolution XPS spectra of Ce 3d, Fe 3p, Ni 2p, C 1s and O 1s.

Utilizing high-sensitivity surface analysis through X-ray photoelectron spectroscopy (XPS) testing, the sample surface's electronic, elemental, and oxidation states were determined. In Fig. 2. XPS measurements were executed for NiO/Ni@C-Fe_3_O_4_/CeO_2_ catalysts to investigate the chemical states and determine their relative proportion of Ni 2p, O 1s, Fe 3p, and Ce 3d. The high-resolution Ce 3d spectrum showcases eight distinctive peaks resulting from the spin–orbit splitting of Ce 3d_5/2_ and Ce 3d_3/2_, signifying the presence of both Ce^3+^ and Ce^4+^ oxidation states in CeO_2_ due to this spin doublet splitting phenomenon^20^. The peaks at binding energies (BE) 882.3, 889.2, and 897.7 eV are assigned to Ce 3d_5/2_, whereas those at 900.8, 907.6, and 917 eV correspond to Ce 3d_3/2_ ionization, highlighting the prevalence of Ce^4+^ 3d states as the predominant valence state in the sample. Additionally, the peaks at 883.9 eV (3d_5/2_ level) and 902.5 eV (3d_3/2_ level) provide compelling evidence supporting the presence of Ce^3+^ 3d final states (19,20). This comprehensive analysis underscores the intricate interplay of Ce oxidation states within the CeO_2_ sample, revealing a nuanced electronic structure^20–22^. The binding energies of Fe 2p_3/2_ and Fe 2p_1/2_ spin–orbit peaks were observed at approximately 711.4 eV and 724.2 eV, respectively. These values closely matched the data obtained for Fe_3_O_4_, confirming the chemical state of iron^23^. Therefore, From the high-resolution spectra of Ce and Fe, the structure of Fe_3_O_4_/CeO_2_ was confirmed.

Notably, the Ni 2p_3/2_ XPS spectra illustrates two peaks at 856.2 and 866.2 eV correspond to the characteristic Ni^2+^ 2p_3/2_ feature, which aligns well with the Ni^2+^ peaks seen in the NiO phase. The peak in binding energy at 853.0 eV could be linked to Ni^0^, confirming the presence of both Ni and NiO on the surface of NiO/Ni^24^. The C 1s peaks of the NiO/Ni@C surface appear at 285.0 and 287.6 eV, which correspond to the C=C and C–O bonds, respectively. Importantly, The O 1s pattern of NiO/Ni@C and Fe_3_O_4_/CeO_2_ surface are also provided. The characteristic binding energy values of 529.7, 531.3, 532.6, and 534.0 eV are assigned to lattice oxygen, oxygen vacancies, adsorbed oxygen, and the C–O–C bond, respectively^20,24^.

### Field-emission scanning electron microscopy (FE-SEM) analysis

**Figure 3:**
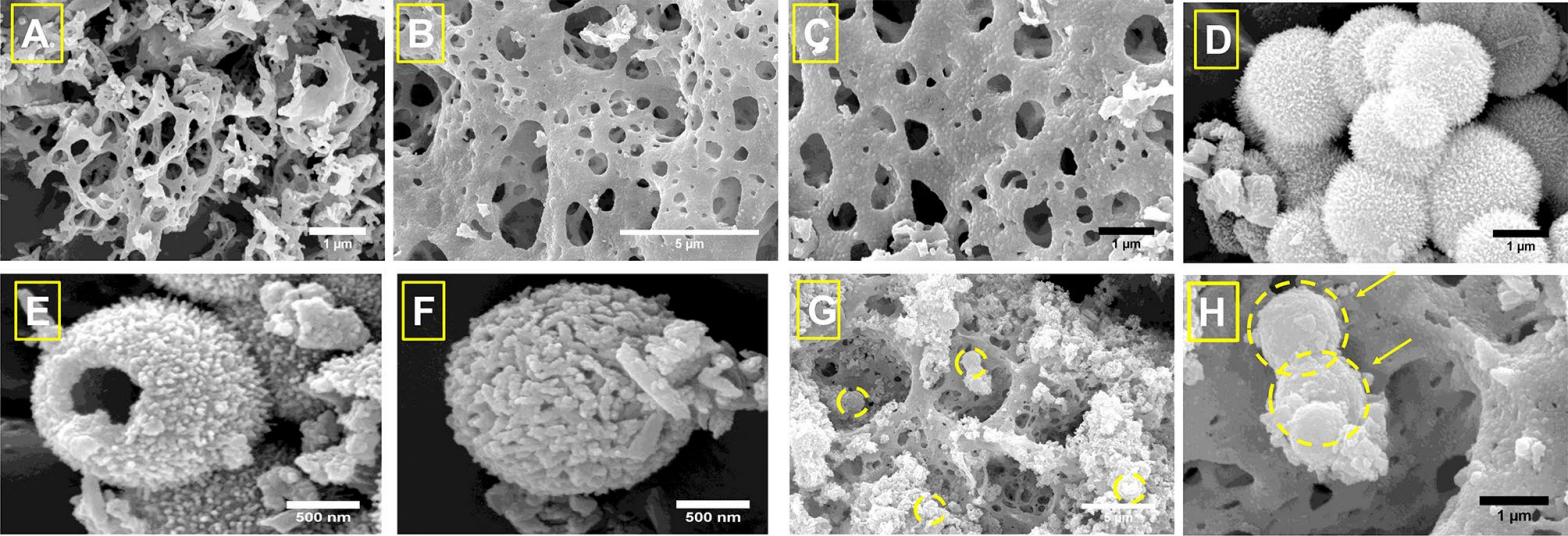
FE-SEM images of (**A**) CeO_2_, (**B**,**C**) Fe_3_O_4_/CeO_2_, (**D**,**E**) Ni-BTC, (**F**) NiO/Ni@C, (**G**,**H**) NiO/Ni@C-Fe_3_O_4_/CeO_2_.

To investigate the morphology of electrocatalyst structures, FE-SEM images were employed. The obtained images in Fig. 3. represent the FE-SEM images corresponding to CeO_2_, Fe_3_O_4_/CeO_2_, Ni-BTC, NiO/Ni@C, and NiO/Ni@C-Fe_3_O_4_/CeO_2_. As displayed in Fig. 3A,B, CeO_2_, as well as Fe_3_O_4_/CeO_2_, respectively, exhibit a remarkably porous and sponge-like structure, indicating a notably large surface area. Additionally, the rough texture observed on the surface of Fe_3_O_4_/CeO_2_ (B), confirms the presence of Fe_3_O_4_ nanoparticles. In Fig. 3C. Ni-BTC displays a uniform spherical structure, with microsphere diameters of approximately 1 to 2 µm. Furthermore, Fig. 3D reveals the hollow structure of a fractured Ni-BTC microsphere. Figure 3E displays the spherical structure of NiO/Ni@C following the calcination of Ni-BTC. Here, the structural morphology remains well-preserved, albeit with greater subface’s roughness and slightly smaller sphere dimensions, averaging around 200 nm or less. Figure 3F affirms the existence of the NiO/Ni@C electrocatalyst on the Fe_3_O_4_/CeO_2_ support, validating the successful synthesis of the porous NiO/Ni@C-Fe_3_O_4_/CeO_2_ nanocomposite.

### Transmission electron microscopy (TEM) analysis

**Figure 4:**
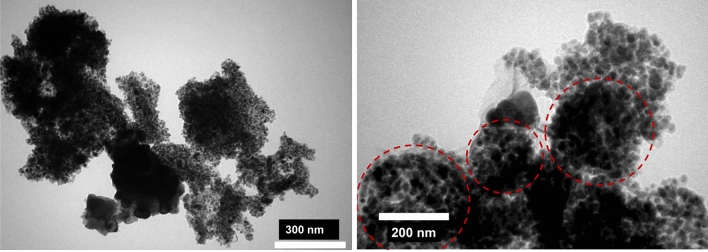
TEM image of NiO/Ni@C-Fe_3_O_4_/CeO_2_ in different magnification.

The TEM image of the NiO/Ni@C-Fe_3_O_4_/CeO_2_ nanocomposite is provided to demonstrate its morphology in Fig. 4. According to this figure, the NiO/Ni@C microspheres (darker areas) are well observable on the Fe_3_O_4_/CeO_2_ oxide support. Furthermore, the image displays the presence of Fe_3_O_4_ nanoparticles. This TEM image aligns well with the FE-SEM image of the same nanocomposite.

### Energy dispersive X-ray (EDX) spectroscopy and elemental mapping

**Figure 5:**
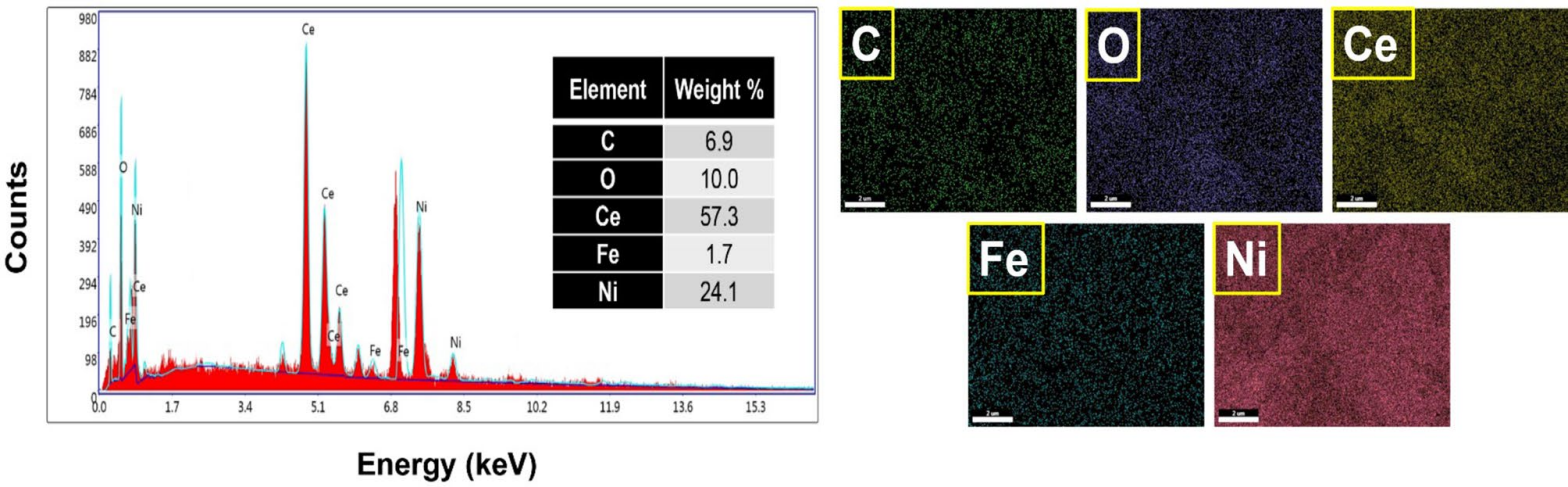
EDX spectrum and elemental mapping of NiO/Ni@C-Fe_3_O_4_/CeO_2_ nanocomposite.

The EDX technique was employed to examine the constituents of the synthesized NiO/Ni@C-Fe_3_O_4_/CeO_2_ nanocomposite. The spectrum displays the presence of Ce, Fe, Ni, O, and C elements. Additionally, elemental mapping indicates the distribution of these elements in the nanocomposite's structure. Figure 5 demonstrates the results of these two analyses.

### Electrochemical assessment

**Figure 6:**
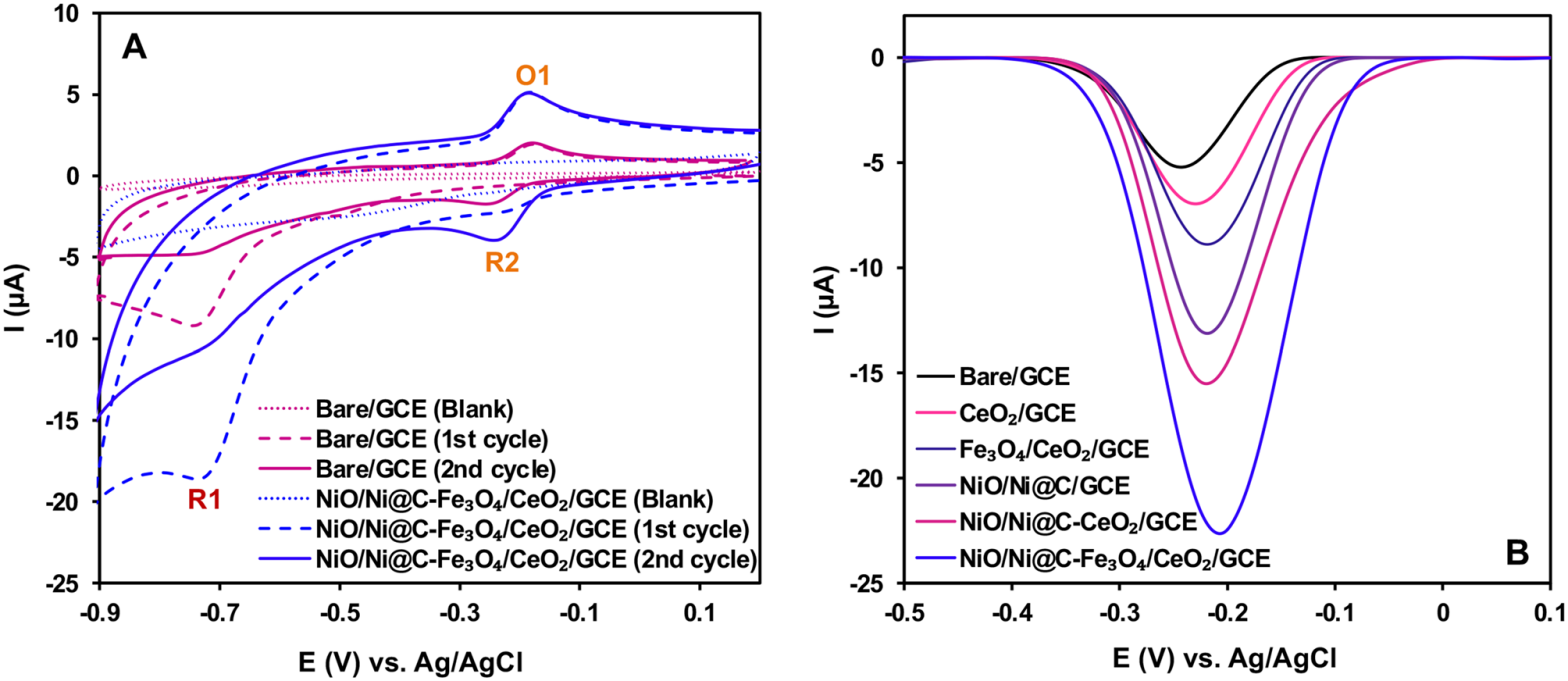
(**A**) CV at a scan rate of 100 mV s^−1^ and (**B**) DPV (t_p_ = 40 ms and H_p_ = 80 mV) curves of bare GCE, CeO_2_/GCE, Fe_3_O_4_/CeO_2_/GCE, NiO/Ni@C/GCE, NiO/Ni@C-CeO_2_/GCE, and NiO/Ni@C-Fe_3_O_4_/CeO_2_/GCE in 2.0 μM NA for CV and DPV in 0.10 M PBS at pH 7.4.

Three electrochemical techniques, including cyclic voltammetry (CV), differential pulse voltammetry (DPV), and electrochemical impedance spectroscopy (EIS), are employed to study the electrochemical behaviours of NA. Figure 6A illustrates CV curves of 2.0 μM NA at the bare GCE, and NiO/Ni@C-Fe_3_O_4_/CeO_2_/GCE in 0.1 M PBS (pH = 7.4, Potential range: (− 0.9)–(− 0.2) V, Scan rate = 100 mV s^−1^). As indicated here, each curve highlights an irreversible reduction peak (R1), closely associated with the irreversible conversion of the nitro group into the hydroxylamine group. The subsequent set of reduction–oxidation peaks (O1 and R2) displays the electrochemical interaction between the hydroxylamine and the nitroso groups. Noticeably, the unmodified GCE sensor's CV curve shows weak reversible peaks, indicating a limited electrochemical response. On the other hand, the NiO/Ni@C-Fe_3_O_4_/CeO_2_/GCE sensor showcases an enhanced electrochemical response, leading to elevated peak current values. This improvement can be attributed to the reason that NiO/Ni@C and Fe_3_O_4_/CeO_2_ improved the conductive properties, the surface area, the number of active reaction sites, and the porosity, which subsequently enhanced the electron transfer kinetics and the adsorption of NA on NiO/Ni@C-Fe_3_O_4_/CeO_2_/GCE. Besides, in Fig. 6B DPV curves were utilized to evaluate the effectiveness of bare GCE, CeO_2_/GCE, Fe_3_O_4_/CeO_2_/GCE, NiO/Ni@C/GCE, NiO/Ni@C-CeO_2_/GCE, and NiO/Ni@C-Fe_3_O_4_/CeO_2_/GCE sensors in detecting NA, which are perfectly aligned with the previously mentioned findings. Hence, selecting NiO/Ni@C-Fe_3_O_4_/CeO_2_/GCE is a favorable choice for conducting additional research regarding NA detection.

EIS was utilized to explore the electron transfer characteristics of the fabricated sensors. Figure S1 demonstrates the Nyquist plots for the bare GCE, CeO_2_/GCE, Fe_3_O_4_/CeO_2_/GCE, NiO/Ni@C/GCE, NiO/Ni@C-CeO_2_/GCE, and NiO/Ni@C-Fe_3_O_4_/CeO_2_/GCE in a solution containing 10 mM [Fe(CN)_6_]^3−/4−^ and 0.1 M KNO_3_, with a frequency range from 250 mHz to 50 kHz, at a potential of 0.2 V. The electrochemical performance of these sensors is closely related to the electron transfer resistance (R_ct_), which becomes evident through the presence of the semicircle's diameter. It can be observed that the bare GCE exhibits the highest R_ct_ value owing to its inferior conductivity characteristics. By contrast, NiO/Ni@C-Fe_3_O_4_/CeO_2_/GCE’s semicircular diameter observed in the high-frequency region is less extensive compared to the other electrodes. This result indicates that the fabricated sensor boosts electrical conductivity and exhibits significantly improved electrochemical performance compared to the unmodified GCE sensor.

Figure S2 displays the corresponding fitting correlation between peak current and scan rate. Noticeable linear relationships are apparent between peak currents and the square root of the scan rate of bare GCE, CeO_2_/GCE, Fe_3_O_4_/CeO_2_/GCE, NiO/Ni@C/GCE, NiO/Ni@C-CeO_2_/GCE, and NiO/Ni@C-Fe_3_O_4_/CeO_2_/GCE in a solution containing 10 mM [Fe(CN)_6_]^3−/4−^ and 0.1 M KNO_3_ at a scan rate of 50 mV s^−1^. The electrochemically active surface area (ECSA) can be determined using the Randles–Sevcik equation (Eq. 1).

1 $${i}_{p}=2.69 \times {10}^{5}{n}^\frac{3}{2}A{D}^\frac{1}{2}C{\upsilon }^\frac{1}{2}$$

In this formula, "*n*" corresponds to the number of electrons participating in the redox reaction, "*i*_*p*_" represents the oxidation peak current (*A*), "*D*" indicates the diffusion coefficient, measured at a value of 8.3 × 10^−6^ cm^2^/s, "*A*" represents the effective electrochemical surface area (cm^2^), "*υ*" signifies the scan rate (V/s), and "*C*" stands for the concentration in the bulk solution, (10 μmol/cm^3^).

The ECSA of bare GCE, CeO_2_/GCE, Fe_3_O_4_/CeO_2_/GCE, NiO/Ni@C/GCE, NiO/Ni@C-CeO_2_/GCE, and NiO/Ni@C-Fe_3_O_4_/CeO_2_/GCE sensors were determined to be 0.013, 0.033, 0.036, 0.067, 0.099 and 0.118 cm^2^, respectively. Therefore, the final fabricated sensor exhibits the largest ECSA, which can be attributed to the increase in the number of active reaction sites and the expansion of the surface area, which is aligned with earlier results.

### Optimization of the experimental process

#### Impact of pH value

**Figure 7:**
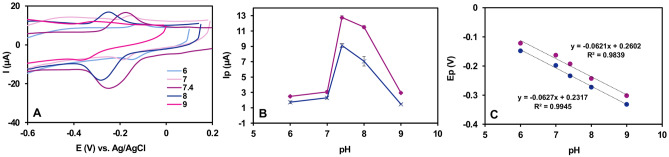
(**A**) Influence of pH (6.0–9.0) on the electrochemical behaviour of NA at NiO/Ni@C-Fe_3_O_4_/CeO_2_/GCE using CV at scan rates of 100 mV s^−1^; (**B**) influence of pH on the peak current of NA; and (C) dependence of the oxidation and reduction peaks potential of NA with pH. Conditions: 2.0 μM NA in 0.1 M PBS.

Figure 7A demonstrates the effect of different pH values on the DPV curves of 2.0 μM NA at the NiO/Ni@C-Fe_3_O_4_/CeO_2_/GCE in a 0.1 M PBS solution (pH = 6.0–9.0). As can be seen in Fig. 7B The electrochemical response of NA exhibited an initial rise followed by a subsequent decline with increasing pH to the peak value as the pH value reached 7.4. Subsequently, the DPV peak current experiences a gradual reduction. This phenomenon can be explained by the fact that the highly acidic environment is not conducive to the electrochemical conversion from –NHOH to –NO, whereas the low concentration of H^+^ ions promotes the degradation of NA, leading to an adverse influence on the irreversible reaction of NA^25^. As depicted in Fig. 7C, it is possible to establish a favorable linear fit between the peak potential (*E*_*p*_) and pH value using the regression equations *E*_*pa*_ =  *− 0.0621 pH* + *0.2607* (*R* ^*2*^ = *0.984*) and *E*_*pc*_ =  *− 0.0627 pH* + *0.2317* (*R* ^*2*^ = *0.995*). The resulting gradients closely approximate the expected value of Nernstian response (59.2 mV pH^−1^). This observation implies that the redox reaction of NA entails an equal participation of protons and electrons. In accordance with the formula provided below:

2 $${dE}_{p}/dpH=2.303mRT/nF$$

In an electrochemical reaction, where "*n*" is the number of transferred electrons and "*m*" represents the number of protons engaged in the process, the ratios of "*m/n*" were approximately 1 for the modified electrode. Therefore, the electrochemical redox of NA for fabricated electrodes should involve a two-electron and two-proton mechanism, which aligns with previously reported observations.

#### Electrochemical reaction mechanism of NA

**Figure 8:**
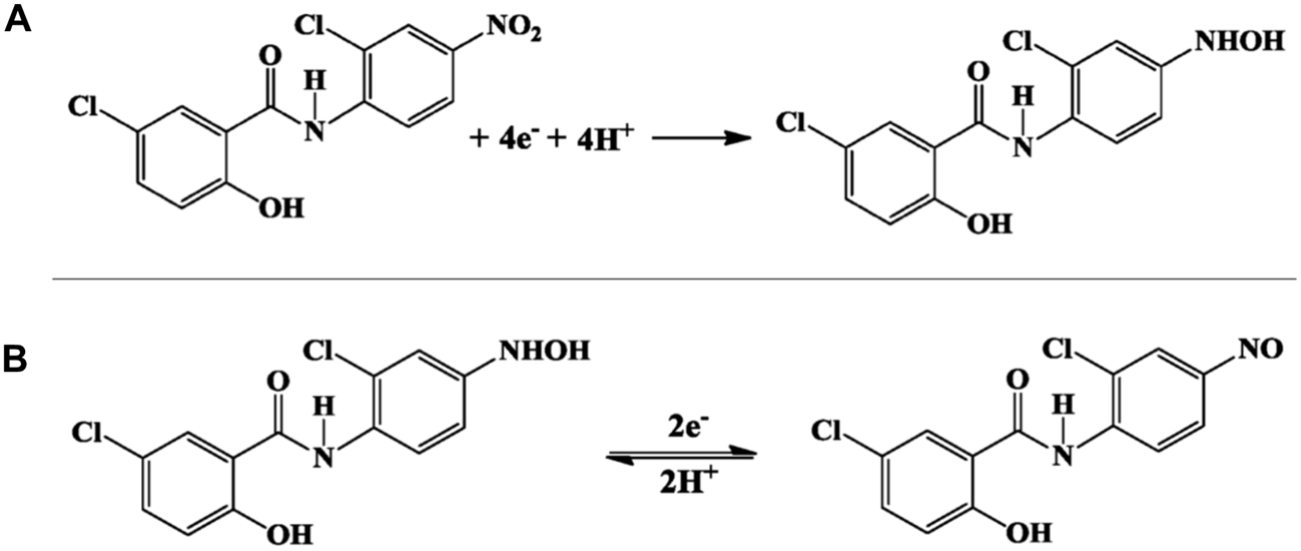
Proposed mechanism for electrochemical behaviours of NA^26^.

In the majority of previously reported publications, the electrochemical response of nitroaromatic compounds typically exhibited two reduction peaks alongside a singular oxidation peak. As shown in Fig. 8A, the irreversible reduction process was connected to the transformation of nitro groups into hydroxylamine species through a four-electron reduction mechanism. In contrast, the reversible redox peaks were attributed to a two-electron transfer, during which electrochemically generated hydroxylamine species transformed into nitrosamine groups (Fig. 8B).

#### The influence of scan rates

**Figure 9:**
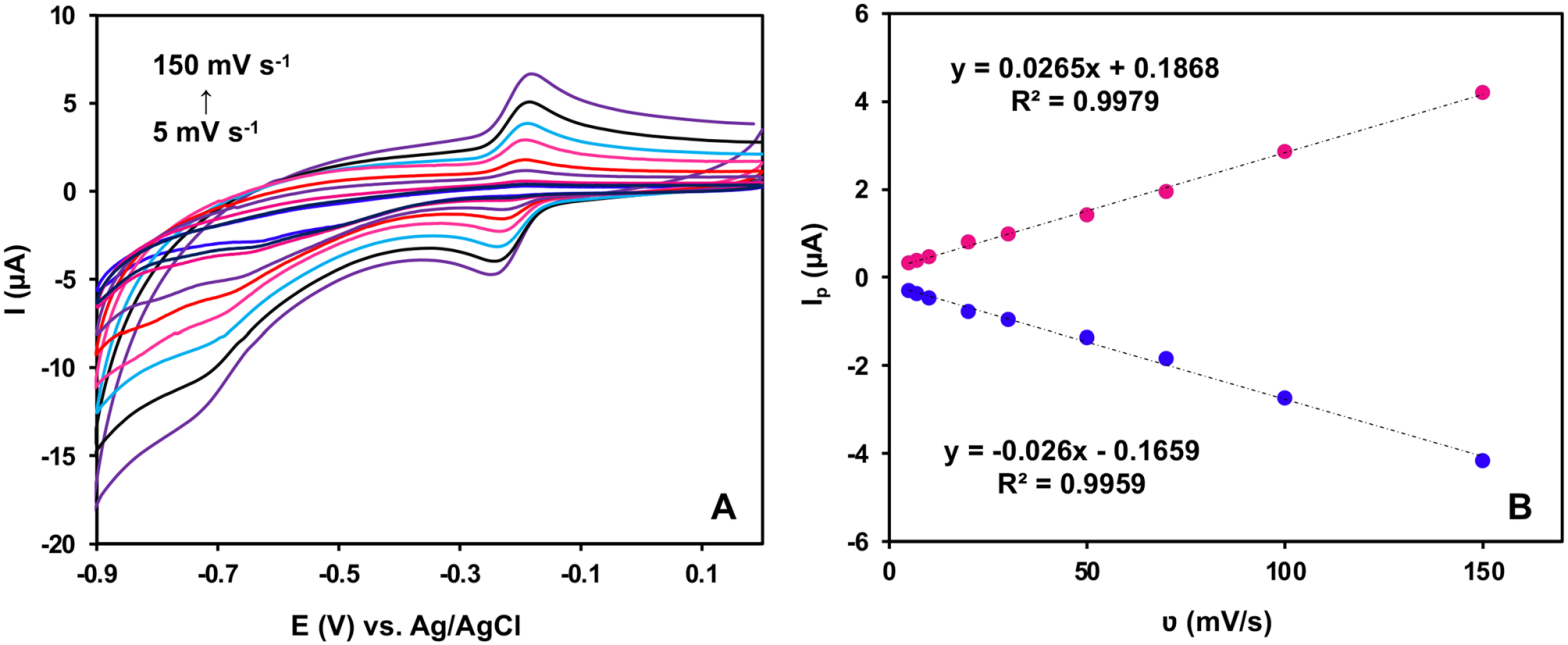
(**A**) Influence of various scan rates (5–150 mV/s) on the detection performance of 2.0 μM NA at the NiO/Ni@C-Fe_3_O_4_/CeO_2_/GCE sensor, and (**B**) linear fitting relationship between peak current and scan rate (0.10 M PBS at pH 7.4).

Figure 9A illustrates the influence of various scan rates (from 5 to 150 mV/s) on the CV during the detection of 2.0 μM NA utilizing the NiO/Ni@C-Fe_3_O_4_/CeO_2_/GCE sensor. It is evident that the scan rate significantly affects the electrochemical response in NA detection. With increasing scan rates, the CV curve's peak current gradually rises. The results reveal that varying the scan rate significantly influences the electrochemical response in NA detection. As the scan rate rises, the peak current within the CV curve shows a noticeable gradual increase. Figure 9B establishes a linear relationship between peak current (*I*_*p*_) and scan rate (*v*). The regression equations can be expressed as *I*_*pa*_ = *0.0265v* + *0.1868* (*R* ^*2*^ = *0.998*) and *I*_*pc*_ =  *− 0.026v – 0.026* (*R* ^*2*^ = *0.9959*). This assessment suggests a strong linear correlation between peak current and scan rate, indicating a close association between the electrochemical detection of NA using the fabricated sensor and a process predominantly governed by adsorption.

#### Accumulation potential and preconcentration time

As illustrated in Fig. S3A, the effect of potential at the NiO/Ni@C-Fe_3_O_4_/CeO_2_/GCE sensor for 0.10 μM NA reduction was examined within the range of − 0.40 V to + 0.40 V. Initially, the peak current for NA increased as the accumulation potential was significantly raised, reaching its peak at 0.00 V, after which it remained relatively stable. Consequently, 0.00 V was chosen as the ideal accumulation potential for the subsequent experiments. Figure S3B shows the impact of preconcentration time on the voltammetric reactions for 2.0 μM niclosamide at a potential of 0.00 V. The peak currents increased up to 180 s, after which no substantial change in the peak current was observed. Consequently, a preconcentration time of 180 s was chosen for all subsequent measurements.

#### Optimization of experimental parameters

As shown in Fig. S4, the impact of the amount of NiO/Ni@C-Fe_3_O_4_/CeO_2_ on the electrochemical response of 0.1 μM NA at NiO/Ni@C-Fe_3_O_4_/CeO_2_/GCE within a concentration range of 1–4 μg was investigated using DPV (0.1 M PBS, pH = 7.4, t_p_ = 40 ms, H_p_ = 80 ms, t_pc_ = 180 s, and E_pc_ = 0.0 V). The reduction peak of NA on the fabricated sensor reached its maximum at 3 μg of NiO/Ni@C-Fe_3_O_4_/CeO_2_ and gradually decreased. Therefore, the 3 μg amount of NiO/Ni@C-Fe_3_O_4_/CeO_2_ is favourable for NA detection.

Obtaining the optimal balance between minimal noise and maximal signal strength relies on adjusting the pulse amplitude and pulse duration in DPVs. Under optimal conditions, the following parameters have been fine-tuned: 0.2 μM of NA in a 0.1 M PBS solution with a pH of 7.4. Additionally, specific values of 80 mV for pulse amplitude and 40 ms for pulse time have been selected.

#### Analytical performance

**Figure 10:**
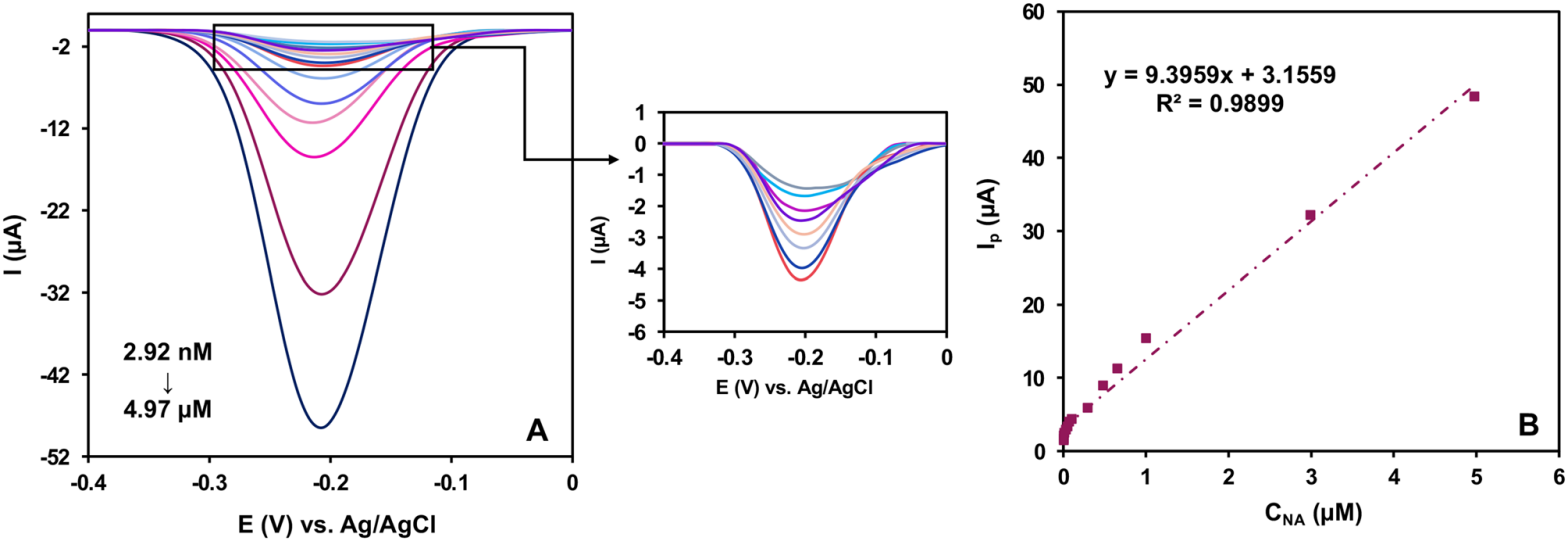
(**A**) DPV profiles at different concentrations of NA (9.0 nM and 30.0 μM) and (**B**) A linear regression relationship between the DPV peak potential of NiO/Ni@C-Fe_3_O_4_/CeO_2_/GCE and the concentration of NA in 0.10 M PBS at pH 7.4 (t_p_ = 40 ms and H_p_ = 80 ms).

NA was evaluated at NiO/Ni@C-Fe_3_O_4_/CeO_2_/GCE under the optimized parameters using DPV. As shown in Fig. 10A, the reduction peak intensity increases as the concentration of NA gradually rises. Figure 10B illustrates a linear relationship between *I*_*p(NA)*_ and NA concentration. The equation representing the linear regression for NA was formulated as *I*_*p*_ = *9.3959C*_*NA(μM)*_ + *3.1559* (*R* ^*2*^ = *0.9899*). According to the calculation results, an impressively low limit of detection (LOD) of 0.91 nM can be achieved. The LOD was determined using the formula which is LOD = 3.3(*S*_bl_/m), where *S*_*bl*_ represents the standard deviations of three reference samples, and *m* stands for the slope of the calibration curves on the NiO/Ni@C-Fe_3_O_4_/CeO_2_/GCE sensor within a linear range of 2.92 nM to 4.97 μM for NA concentration. The Limit of Quantification (LOQ) is the lowest analyte concentration that can be quantitatively detected with a stated accuracy and precision, here is 2.92 nM. Furthermore, the sensitivity was calculated 79.58 μM μA^−1^ cm^−2^.

#### Repeatability, reproducibility, and stability

The repeatability of the designed sensor was assessed by employing 0.10 μM NA in 0.1 M PBS for three consecutive measurements conducted on the surface of NiO/Ni@C-Fe_3_O_4_/CeO_2_/GCE. The obtained results exhibited strong consistency with an RSD of 3.1% (Fig. S5A). Furthermore, the evaluation of reproducibility entailed the fabrication of five distinct NiO/Ni@C-Fe_3_O_4_/CeO_2_/GCE samples under similar conditions, resulting in an observed RSD of 4.8% (Fig. S5B). Finally, the enduring performance of the NiO/Ni@C-Fe_3_O_4_/CeO_2_/GCE in the analysis of NA was investigated by measuring the reduction peak current of NA after 7 days of storage at room temperature. Remarkably, 96% of the initial reduction peak current of NA was preserved (Fig. S5C). These results underscore the notable consistency, reproducibility, and long-term stability of the proposed sensor.

As depicted in Table 1, the comparison of the detection limit and linear range of NA using the NiO/Ni@C-Fe_3_O_4_/CeO_2_/GCE sensor suggests its performance relative to other sensors designed for the detection of NA.

**Table 1:** Comparison between other fabricated electrochemical sensors to determine NA with this study.

Fabricated sensor	Method	Linear range (µM)	Detection limit (nM)
PNRs/SPCNPs-g-CNTs/GCE^25^	DPV	0.01–10	3.6
GCMCN@CTS/GCE^27^	DPV	0.01–10	1.4
BMCN@Pal/GCE^28^	DPV	0.01–10	7.8
3DHPC@CTS/GCE^7^	DPV	0.01–10	6.7
CNT@CBS@CTS/GCE^29^	DPV	0.01–10	4.3
GR-MWCNTs-COOH/GCE^30^	DPV	0.01–10	3.1
CNH@MWCNTs/ GCE^31^	DPV	0.007–10	2.0
MWCNT/CD/GCE^1^	DPV	0.06–15	19.5
Activated SPCE^32^	SWV	0.06–0.50	1.5
HNTs@VXC-72/GCE^6^	DPV	0.01–1	3.28
Pal-Gr-COOH/GCE^33^	DPASV	0.02–0.99	4.6
NiO/Ni@C-Fe_3_O_4_/CeO_2_/GCE (this study)	DPV	2.92 nM–4.97 µM	0.91

It is noticeable that the NiO/Ni@C-Fe_3_O_4_/CeO_2_/GCE exhibited outstanding analytical performance in detecting NA. Moreover, the materials utilized to fabricate NiO/Ni@C-Fe_3_O_4_/CeO_2_/GCE were both cost-effective and undemanding to prepare. Additionally, this sensor offers several advantages for NA detection, such as the arrangement of this electrocatalyst resulted in a greater specific surface area and a multitude of electroactive sites, and NiO/Ni@C spheres and Fe_3_O_4_/CeO_2_ metal oxide layer work together in a synergistic manner to boost the reduction current.

### Interference study

The ability of the NiO/Ni@C-Fe_3_O_4_/CeO_2_/GCE sensor to resist interference was examined in a 0.1 M PBS solution containing 2.0 μM NA, where various interfering ions (at a concentration 1000 times that of NA) including Na^+^, Cl^−^, K^+^, NO_3_^−^, Ca^2+^ ions, as well as other interfering organic compounds such as Urea, Citric acid, Tartaric acid, Saccharin, l-cysteine and glucose (Fig. S5D). The results indicate that interfering ions and organic compounds do not significantly impact the ability of the sensor to detect NA. Consequently, as prepared, the NiO/Ni@C-Fe_3_O_4_/CeO_2_/GCE sensor demonstrates outstanding resistance to interference when determining NA.

### Real sample analysis

The feasibility of using a GCE modified with NiO/Ni@C-Fe_3_O_4_/CeO_2_ for the detection of niclosamide in human urine and pharmaceutical tablets was investigated using the standard addition method (*n* = *3*), as presented in Fig. S6 and Table 2. The results indicate that the developed sensor achieves satisfactory recoveries between 92 and 102% for different spiked samples, with RSD values ranging from 5.5 to 7.5%. Thus, the proposed sensor demonstrates exceptional accuracy and precision when determining NA in human urine and pharmaceutical tablets.

**Table 2:** Results of the determination of NA in human urine and pharmaceutical tablets (confidence interval = 95% and n = 3).

Sample	Spiked (µg)	Found (µg)	Recovery (RSD) (%)
Urine-one	0.49	0.45 ± 0.04	92 (5.5)
	4.91	4.82 ± 0.18	98 (3.35)
Urine-two	0.49	0.47 ± 0.09	95 (4.20)
	4.91	5.11 ± 0.23	104 (7.20)
Niclosamide’s Tablets	–	513.45 ± 0.34 (mg)	102 (7.15)

## Conclusion

To summarize, a MOF-derived nanocomposite of NiO/Ni@C-Fe_3_O_4_/CeO_2_ was fabricated to develop a voltammetric sensor based on NiO/Ni@C-Fe_3_O_4_/CeO_2_/GCE for determination of NA. CeO_2_ and Fe_3_O_4_ nanoparticles were synthesized using a fast and cost-effective technique, employing an aqueous extract obtained from the peel of the Musa sapientum fruit. Moreover, The Ni-MOF was produced using a facile solvothermal process and subsequently altered into NiO/Ni@C through calcination at 450 °C. The multifunctional collaboration between Fe_3_O_4_/CeO_2_ and NiO/Ni@C greatly enhanced the detection of NA. The structural and morphological attributes of CeO_2_, Fe_3_O_4_, Fe_3_O_4_/CeO_2_, NiO/Ni@C, and NiO/Ni@C-Fe_3_O_4_/CeO_2_ were characterized through a comprehensive analysis involving techniques including FT-IR spectroscopy, XRD, XPS analysis, FE-SEM, TEM, EDS, and elemental mapping. The results unveil an extensive surface area, enhanced electrical conductivity, and numerous reactive sites. Owing to these features, the suggested electrochemical sensor employing NiO/Ni@C-Fe_3_O_4_/CeO_2_ for detecting of NA demonstrates a notable low limit of detection, an extensive operational range, and superb specificity. In other words, it exhibits a linear range from 2.92 nM to 4.97 μM with a low detection limit of 0.91 nM. Concerning reliability, it has successfully ascertained the presence of NA in human urine and pharmaceutical tablets, achieving recovery rates of 92–102% with RSD values of 5.5–7.15%. Therefore, the fabrication of NiO/Ni@C-Fe_3_O_4_/CeO_2_/GCE sensor offers an essential reference for developing electrochemical sensors for NA.

## Supplementary Information

Supplementary Information.

## Data Availability

The datasets supporting the conclusions of this article are included within the article.
