# Supplementary material for: Design and fabrication of electrochemical sensor based on NiO/Ni@C-Fe3O4/CeO2 for the determination of niclosamide
Source: Sci Rep. 2024 Mar 30;14:7576. doi: 10.1038/s41598-024-58319-w (PMC10981725; doi:10.1038/s41598-024-58319-w)
Supplement: Supplementary file 1 — Supplementary Information. [file 41598_2024_58319_MOESM1_ESM.pdf]

# Supplementary Materials

## Design and Fabrication of Electrochemical Sensor Based on NiO/Ni@C-$Fe_3O_4$/$CeO_2$ for The Determination of Niclosamide

**Setayesh Darvishi[1], Ali A. Ensafi*[1,2] Kimia Zarean Mousaabadi [1]**

*1. Department of Chemistry, Isfahan University of Technology, Isfahan 84156-83111, IRAN*

*2. Adjunct Professor, Department of Chemistry & Biochemistry, University of Arkansas, Fayetteville, AR 72701, USA.*

**Experimental section:**

**Apparatus**

Various spectroscopic and analytical techniques were employed to characterize NiO/Ni@C-$Fe_3O_4$/$CeO_2$/GCE. Fourier-transform infrared (FT-IR) spectra were acquired using a 680-plus spectrophotometer. X-ray diffraction (XRD) patterns were generated using an AW-XDM300 X-ray diffractometer (Asenware Co., China). Field emission-scanning electron microscope (FE-SEM) images were captured using a Quanta 450 FEG scanning electron microscopy instrument (FEI Co., USA), operating at an acceleration voltage of 25 kV, and equipped with map analysis and energy-dispersive X-ray analysis (EDAX-Octane Elite). Transmission electron microscopy (TEM) analysis was performed using a Philips CM120 instrument (Philips Co., Netherlands). The Brunauer-Emmet-Teller (BET) surface area was measured using a BELSORP MINI II surface area analyzer (BEL Co., Japan) through N2 adsorption-desorption analysis. Atomic force microscopy (AFM), utilizing an ENTEGRA instrument (NT–MDT, Moscow), was employed for further characterization. Electrochemical measurements were conducted using an Autolab

potentiostat/galvanostat (PGSTAT101) system. Additionally, the working, reference, and counter electrodes consisted of a modified GCE, an Ag/AgCl/KCl 3.0 M electrode, and Pt electrodes, respectively.

**Modified electrode preparation**

The unmodified GCE underwent a 3-minute polishing procedure using 0.05 μm alumina powders, followed by a 3-minute sonication process in a mixture of ethanol and water (in a 1:1 volume ratio) to ensure surface cleanliness. Subsequently, an optimal volume of well-mixed NiO/Ni@C-$Fe_3O_4$/$CeO_2$ suspension was applied to the surface of the working electrode and allowed to air-dry at room temperature.

**Preparation of real sample**

This study collected urine samples from fasting individuals and immediately stored them in a refrigerator. Subsequently, niclosamide was added to the collected samples. In the next step, the mixture was centrifuged at 12,000 revolutions per minute for 15 minutes to remove suspended particles. In the following stage, dilution was performed using a buffer solution with a pH of 4.7 to achieve the desired concentration.

Initially, two 500 mg niclosamide tablets were ground in an agate mortar as a sample for the niclosamide tablets. They were then dissolved in ethanol and subjected to ultrasonic bath treatment for 16 minutes. In the next step, they were centrifuged at 12,000 revolutions per minute for 15 minutes. Finally, dilution was performed using a buffer solution with a pH of 4.7 to reach the desired concentration.

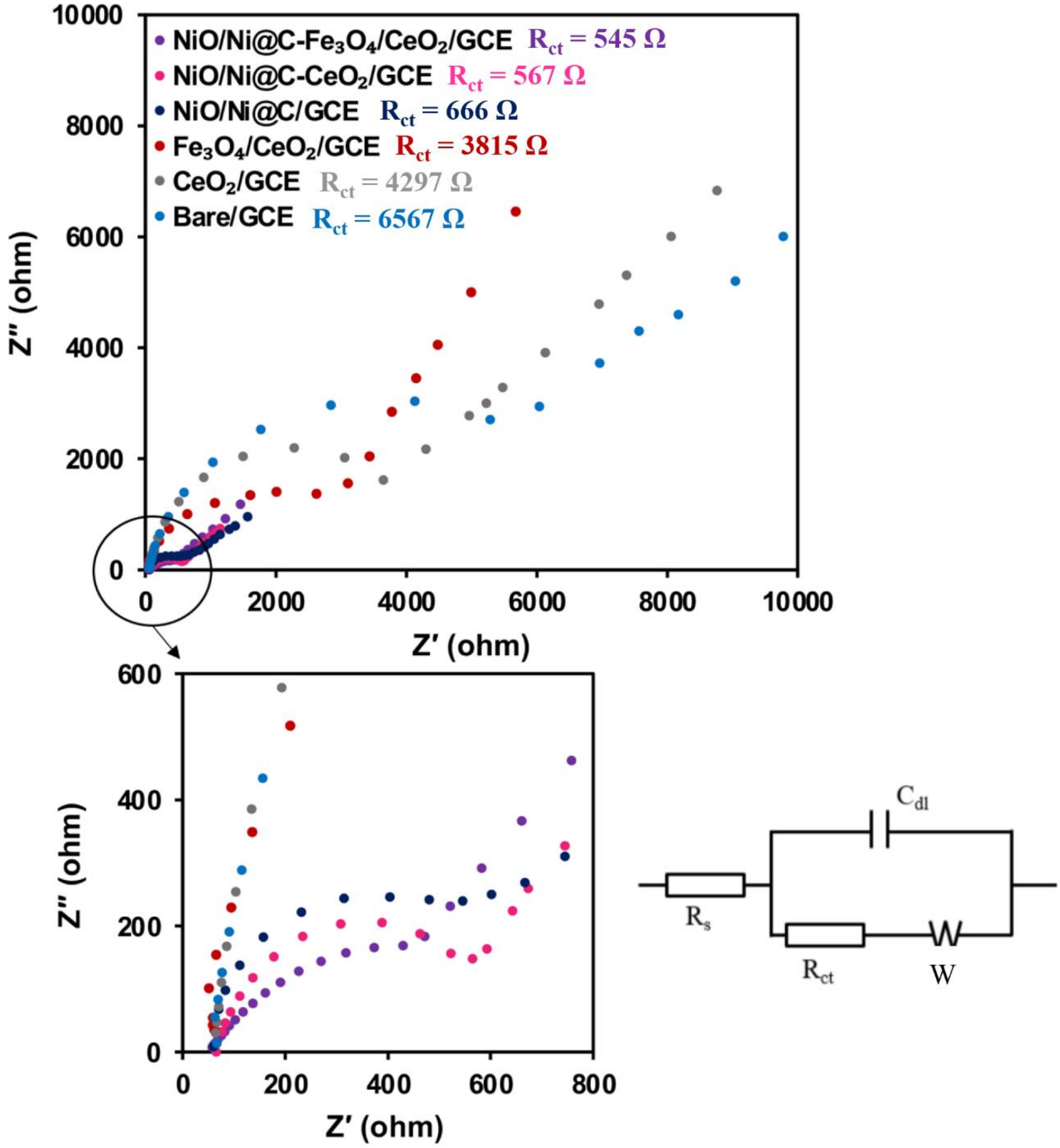


**Figure S1.** Impedance spectra of bare GCE, $CeO_2$/GCE, $Fe_3O_4$/$CeO_2$/GCE, NiO/Ni@C/GCE, NiO/Ni@C-$CeO_2$/GCE and NiO/Ni@C-$Fe_3O_4$/$CeO_2$/GCE, and the equivalent circuit of NiO/Ni@C-$Fe_3O_4$/$CeO_2$/GCE.

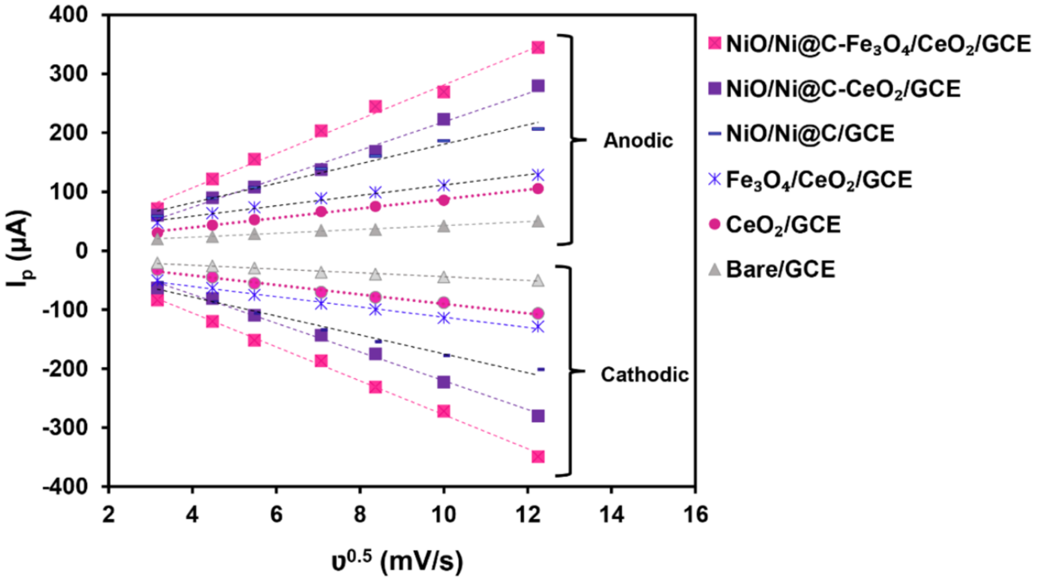


**Figure S2.** The linear fitting relationship of bare GCE, $CeO_2$/GCE, $Fe_3O_4/CeO_2$/GCE, NiO/Ni@C/GCE, NiO/Ni@C-$CeO_2$/GCE, and NiO/Ni@C-$Fe_3O_4/CeO_2$/GCE in 10 mM $[Fe(CN)_6]^{3-/4-}$ and 0.1 M $KNO_3$ at a scan rate of 50 mV $s^{-1}$.

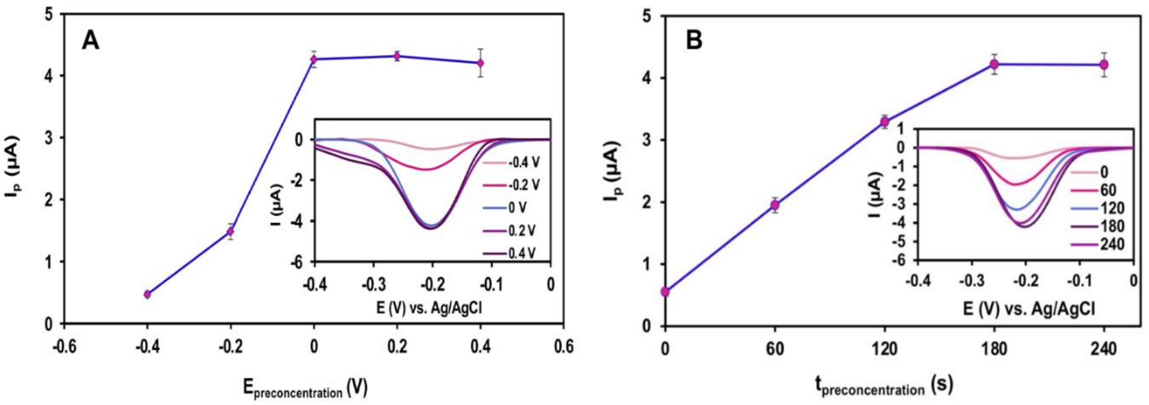


**Figure S3.** Dependence of the peak current with different preconcentration (A) potential, (B) time (0.1 $\mu$M, 0.1M PBS, pH = 7.4, $t_p$= 40 ms, $H_p$ = 80 ms, $t_{pc}$= 180 s, and $E_{pc}$= 0.0 V).

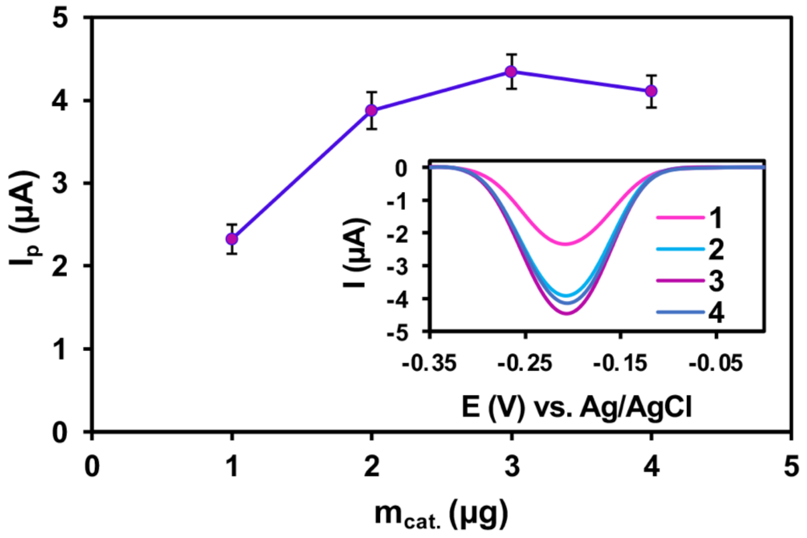


**Figure S4.** The relationship between the amount of the NiO/Ni@C-$Fe_3O_4$/$CeO_2$ and DPV reduction peak response value (0.1 μM, 0.1 M PBS, pH = 7.4, $t_p$= 40 ms, $H_p$ = 80 ms, $t_{pc}$= 180 s, and $E_{pc}$= 0.0 V).

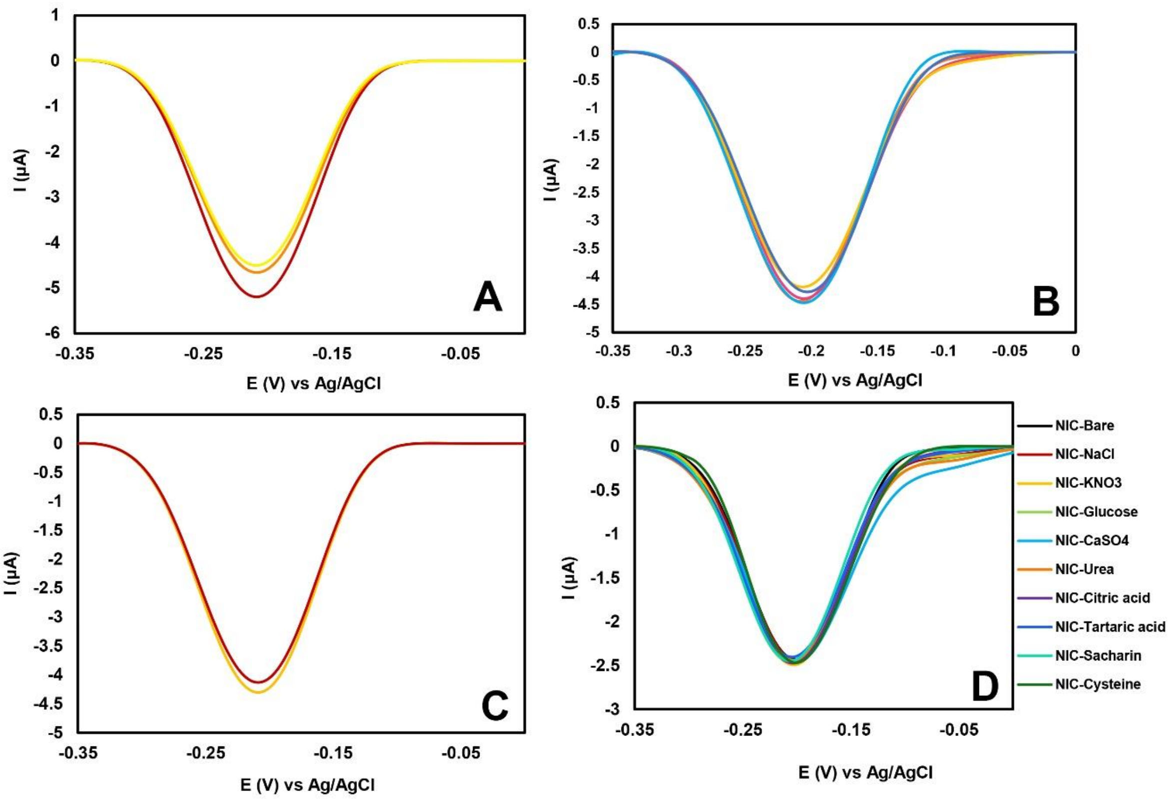


**Figure S5.** DPV reduction peak response value under optimal conditions for (A) Repeatability (n = 3), (B) Reproducibility (n = 5), (C) stability, and (D) Interference study.

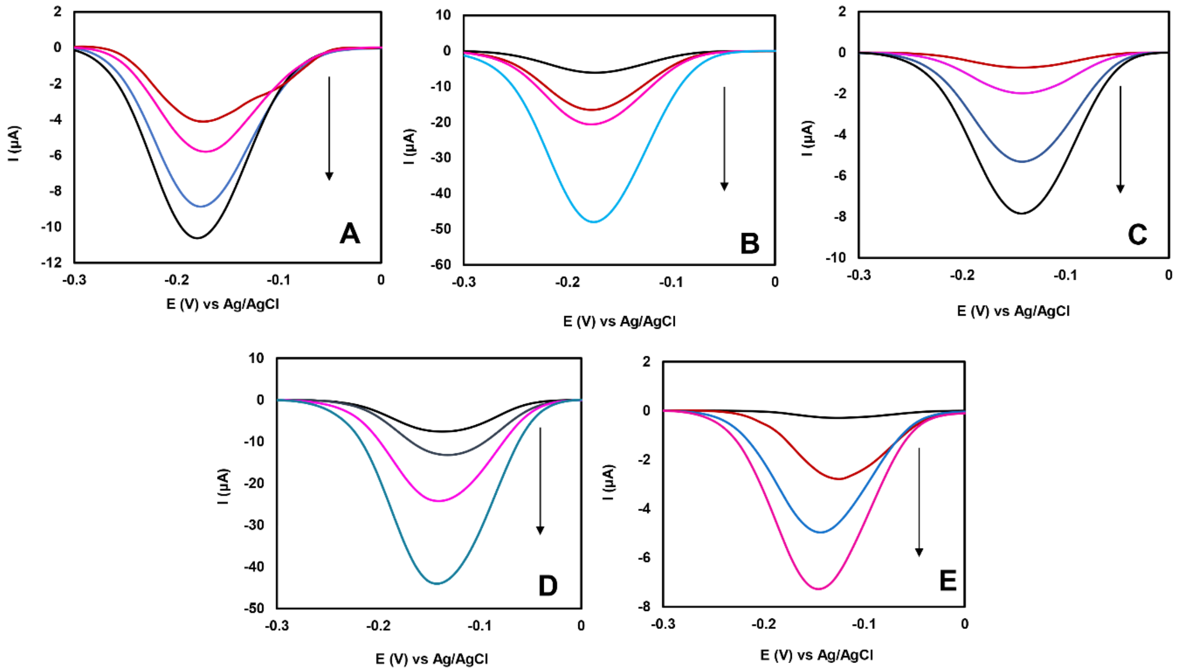


**Figure S6.** DPV graphs for real analysis (A) Urine-one (spiked = 0.49 µg), (B) Urine-one (spiked = 4.91 µg), (C) Urine-two (spiked = 0.49 µg), (D) Urine-two (spiked = 4.91 µg), and (E) Niclosamide's Tablets.
